# Persistence and dispersal in a Southern Hemisphere glaciated landscape: the phylogeography of the spotted snow skink (*Niveoscincus ocellatus*) in Tasmania

**DOI:** 10.1186/s12862-015-0397-y

**Published:** 2015-06-26

**Authors:** H.B. Cliff, E. Wapstra, C.P. Burridge

**Affiliations:** School of Biological Sciences, University of Tasmania, Private Bag 55, Hobart, Tasmania 7001 Australia

**Keywords:** Glacial refugia, Pleistocene, Tasmania, Phylogeography, Reptile, Recolonisation

## Abstract

**Background:**

The aim of this research was to identify the effects of Pleistocene climate change on the distribution of fauna in Tasmania, and contrast this with biotic responses in other temperate regions in the Northern and Southern Hemisphere that experienced glacial activity during this epoch. This was achieved by examining the phylogeographic patterns in a widely distributed Tasmanian endemic reptile, *Niveoscincus ocellatus*. 204 individuals from 29 populations across the distributional range of *N. ocellatus* were surveyed for variation at two mitochondrial genes (ND2, ND4), and two nuclear genes (β-globin, RPS8). Phylogenetic relationships were reconstructed using a range of methods (maximum parsimony, Bayesian inference and haplotype networks), and the demographic histories of populations were assessed (AMOVA, Tajima’s *D,* Fu’s *Fs*, mismatch distributions, extended Bayesian skyline plots, and relaxed random walk analyses).

**Results:**

There was a high degree of mitochondrial haplotype diversity (96 unique haplotypes) and phylogeographic structure, where spatially distinct groups were associated with Tasmania’s Northeast and a large area covering Southeast and Central Tasmania. Phylogeographic structure was also present within each major group, but the degree varied regionally, being highest in the Northeast. Only the Southeastern group had a signature of demographic expansion, occurring during the Pleistocene but post-dating the Last Glacial Maximum. In contrast, nuclear DNA had low levels of variation and a lack of phylogeographic structure, and further loci should be surveyed to corroborate the mitochondrial inferences.

**Conclusions:**

The phylogeographic patterns of *N. ocellatus* indicate Pleistocene range and demographic expansion in *N. ocellatus*, particularly in the Southeast and Central areas of Tasmania. Expansion in Central and Southeastern areas appears to have been more recent in both demographic and spatial contexts, than in Northeast Tasmania, which is consistent with inferences for other taxa of greater stability and persistence in Northeast Tasmania during the Last Glacial Maximum. These phylogeographic patterns indicate contrasting demographic histories of populations in close proximity to areas directly affected by glaciers in the Southern Hemisphere during the LGM.

**Electronic supplementary material:**

The online version of this article (doi:10.1186/s12862-015-0397-y) contains supplementary material, which is available to authorized users.

## Background

The glacial-interglacial oscillations of the Pleistocene have strongly influenced the distribution and evolution of many species [1]. Initial interest in the effects of these oscillations largely focused on temperate species within Europe [2]. The classic phylogeographic view is that these climatic changes forced a diverse range of temperate European taxa to become geographically restricted to a series of spatially discontinuous, low latitude, Mediterranean glacial refugia [1, 3, 4]. During glacial intervals these refugia accumulated genetically divergent lineages, and later provided the source populations for interglacial range expansions. As recolonising populations are expected to represent only a subset of the diversity associated with glacial refugia (due to founder events and high density blocking, see [5]), phylogeographic methods can locate refugia by assessing patterns of genetic diversity across a species’ geographical range [2].

Recently, an increasing number of phylogeographic studies have questioned the simplicity and universality of this classical southern Europe refugia model, describing ‘cryptic’ northern refugia [6–8] and ‘refugia within refugia’ [8, 9] for a range of temperate European species. However, withstanding this growing complexity several facts remain clear—for a large proportion of taxa the LGM had a strong biogeographic influence, and that for most species a limited number of large southern refugia supported the majority of genetic diversity throughout the Pleistocene. While such generalisations can be made for Europe and other Northern Hemisphere regions, the impact of Pleistocene climate change on biota in the Southern Hemisphere remains comparatively understudied [10–15].

**Fig. 1:**
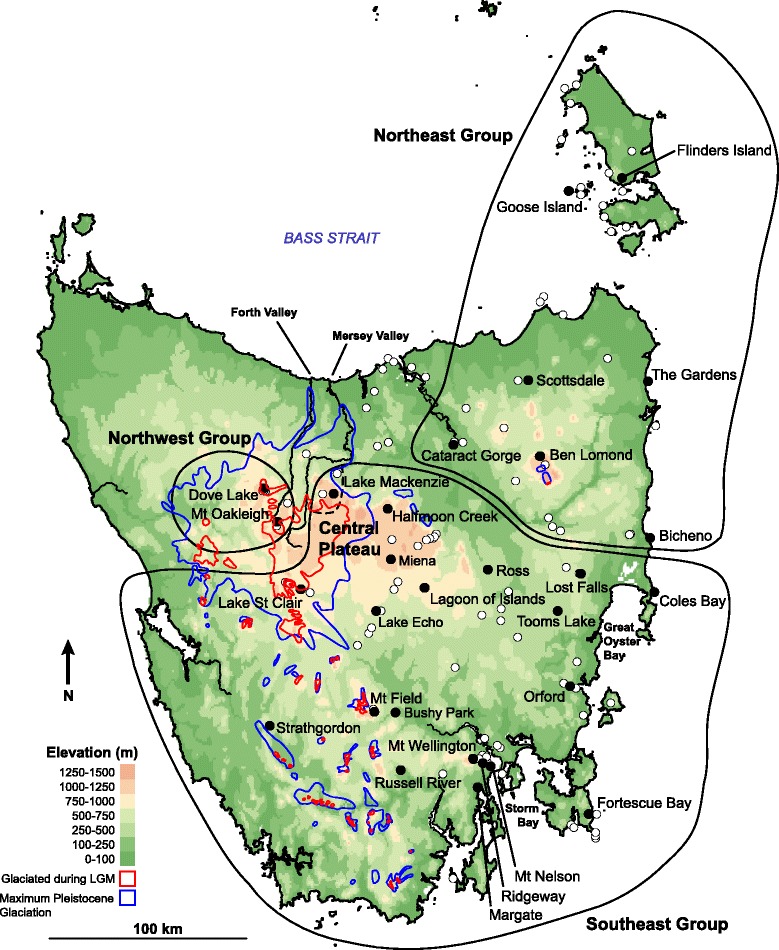
Topographic map of Tasmania indicating sampling sites for *Niveoscincus ocellatus* (filled circles) and species distribution records (open circles). The blue line bounds the maximum extent of Pleistocene glaciation, while that corresponding to the Last Glacial Maximum is bounded by the red line, based on [28]. Black lines demarcate groups defined on the basis of mtDNA variation, with the dashed black line indicating the ambiguous placement of the Lake Mackenzie population either within the Southeast group or on its own. Elevation data were obtained from Geoscience Australia

During glacial periods, ice was absent or discontinuous across most of the Southern Hemisphere continents with the exception of Antarctica. This has meant that many austral studies have focused on the effects of Pleistocene changes in aridity, in tropical or ice-free temperate regions, rather than the effects of glaciers directly (e.g., [16–19]). Furthermore, Southern Hemisphere studies of the impacts of glacial and periglacial activity on species distributions have concentrated on New Zealand, Patagonia, and Antarctica (e.g., [20–24]), while Tasmania—the focus of Australia’s most extensive Pleistocene glacial activity [25]-has been comparatively neglected. Yet, unlike Patagonia and New Zealand, Tasmania has not experienced recent tectonic activity, which has the ability to confound interpretations regarding the influence of glaciations on species distributions and gene flow [22, 24]. Although not presently glaciated, Tasmania possessed glaciers at least five times during the Pleistocene, and these glaciers were most extensive (covering up to 7000 km^2^) during the early (~1.8 Myr) and middle Pleistocene (>130 kyr) [26–30] (Fig. 1). During the LGM, smaller ice caps formed at high altitudes, and local summer temperatures are estimated to have been 6–8 °C cooler than present averages [25, 28, 31] (Fig. 1).

Plant studies indicate a wide range of phylogeographic patterns in Tasmania, but those of several Eucalypt species [32] have led to the suggestion that species responses to Pleistocene glacial activity in Tasmania may be similar to classical European patterns—whereby species retreated into a few large and distinct refugia during the LGM, and the genetic diversity of recolonising populations is limited [33]. However, these patterns contrast strongly with those typically observed in other parts of Australia and the Southern Hemisphere, which have suggested greater numbers of refugia, including many micro-refugia (small pockets of suitable habitat in an otherwise uninhabitable landscape) [34], and genetic structuring reflecting events which pre-date the LGM [17, 18, 22, 24, 35]. Consequently, Tasmania represents an important region for further research to better develop our understanding of the effects of Pleistocene glaciations on species distributions [36]. In this context, lizards have recently been highlighted as good models for use in phylogeographic studies [37]. As ectotherms, lizards are sensitive to changes in climate, which may be manifested in alterations of species distributions [38]. Lizards also typically have low mobility, and phylogeographic patterns from historic events will be retained for longer periods [39].

This study investigated the phylogeography of a Tasmanian endemic reptile, the spotted snow skink (*Niveoscincus ocellatus*). This species is an excellent model for investigating the impacts of Pleistocene glacial cycles in Tasmania for two reasons: it has a wide geographic distribution across Tasmania, and it has clear distribution restrictions associated with climate and habitat type. *Niveoscincus ocellatus* ranges from sea level to high elevations, including previously glaciated regions (Fig. 1) [40, 41], but it is not currently found at altitudes above ~1200 m, beyond which it is sharply replaced by biennially reproducing, alpine specialist species: *N. greeni* and *N. microlepidotus* [42, 43]. Mean temperature for the warmest month is presently ~10 °C at 1200 m elevation, yet the corresponding temperature would have been ~4 °C during the LGM [44]. It is expected that the historical distribution of *N. ocellatus* will have been strongly regulated by Pleistocene climate change, such that there will be genetic signatures of Pleistocene refugia and subsequent range expansion. Furthermore, *N. ocellatus* only occurs amongst rocky outcrops [45], which suggests that gene flow will be generally low [46] (but see [47, 48]) and genetic signatures of historic distributions could be well preserved.

## Results

### Mitochondrial DNA sequence variation and phylogeographic relationships

**Fig. 2:**
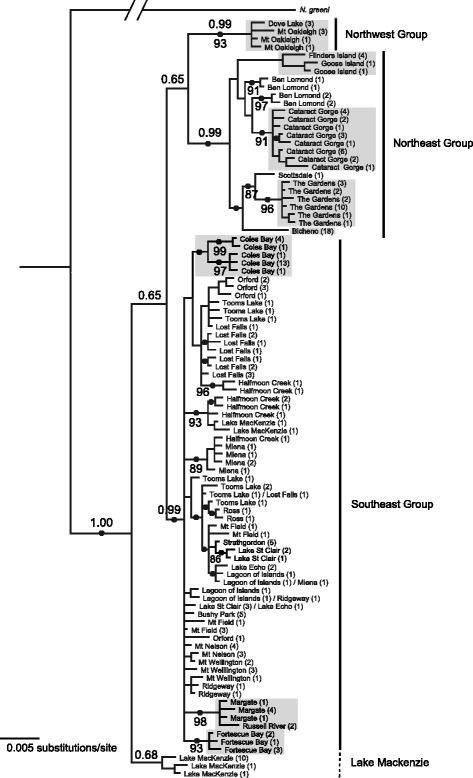
Bayesian inference tree for *Niveoscincus ocellatus* based on 1420 bp of ND2 and ND4 mitochondrial DNA sequence. Branch lengths are scaled relative to the scale bar. Posterior probabilities >0.95 are indicated by the black dots, with values at key nodes also specified above branches at nodes. Bootstrap values from parsimony analysis, where they exceeded 70 %, are also listed below branches. Numbers in parentheses indicate the number of individuals from a site exhibiting that haplotype. The branch leading to the outgroup *N. greeni* was truncated to aid visualisation, and likewise that leading to *N. pretiosus* was removed. Grey boxes highlight regionally monophyletic areas within the major clades

A total of 95 unique haplotypes were identified from the 204 *N. ocellatus* sampled, with 183 (12.9 %) of 1420 characters variable and 147 (10.4 %) parsimony-informative. Tree topologies were very similar between maximum parsimony and Bayesian tree-building methods, only differing in relationships at shallow phylogenetic levels, while the composition of major clades were consistent across analyses; therefore only the Bayesian inference tree is presented (Fig. 2; parsimony tree Additional file 1: Figure S1). Four major clades are evident: a ‘Northeastern’ clade, a ‘Southeastern’ clade, a ‘Northwestern’ clade, and a clade of three Lake Mackenzie haplotypes (Fig. 2). The relationships among these four clades were uncertain and received low topological support, but with the exception of the three Lake Mackenzie haplotypes each clade received posterior probability greater than 0.95. The topology obtained from BEAST analysis using a coalescent tree prior and a strict, externally calibrated, molecular clock was compatible with that described above, and suggests that the major lineages diverged during the last 2 Myr (Additional file 1: Figure S2).

SAMOVA recovered a group corresponding to the Northeastern clade when testing for two populations (Φ_CT_ = 0.50, P = 0.00), and when testing for three populations recovered groups similar to that inferred by the phylogeny (Φ_CT_ = 0.53, P = 0.00): Northeastern, Southeastern (but with inclusion of both Lake McKenzie and Dove Lake), and Northwestern (Mt. Oakleigh only). Given no appreciable increase in Φ_CT_ with increasing number of populations in SAMOVA, three regional groups were defined for further population genetic analyses. The Northeastern and Northwestern (Mt Oakleigh and Dove Lake) clades each corresponded to respective groups, but the Lake Mackenzie clade (three haplotypes) was combined into the same group as the Southeastern clade (hereafter, the Southeastern group) as two of the Lake Mackenzie haplotypes clustered monophyletically among Southeastern haplotypes, and this locality is geographically confluent with Southeastern clade localities. The same three groups are readily distinguished by large numbers (>19) of mutations in the TCS network (Additional file 1: Figure S3). With respect to these groups, AMOVA indicated that spatial structuring of mitochondrial genetic variation was significant at each hierarchical level—among populations, among populations within groups, and among groups (Table 1). The Northeastern group had the highest degree of spatial structuring, where most localities (excluding Ben Lomond) were individually monophyletic (Fig. 2). Structuring was lower within the Southeastern group, where only a few populations of contiguous regions formed monophyletic clades (Table 1; Fig. 2).

**Table 1:** Analysis of molecular variance (AMOVA) comparing structure in groups (Northwest, Northeast, Southeast) as defined based on mtDNA phylogeny

	mtDNA		β-globin	
Source of variation	Fixation index	P-value	Fixation index	P-value
Among groups	0.52 (Φ_CT_)	<0.0001	0.02 (Φ_CT_)	<0.0001
Among populations within groups	0.75 (Φ_SC_)	<0.0001	0.36 (Φ_SC_)	<0.0001
Among all populations	0.88 (Φ_ST_)	<0.0001	0.37 (Φ_ST_)	<0.0001
Among SE group populations	0.63 (Φ_ST_)	<0.0001	0.32 (Φ_ST_)	<0.0001
Among NE group populations	0.89 (Φ_ST_)	<0.0001	0.18 (Φ_ST_)	<0.0001
Among NW group populations	0.43 (Φ_ST_)	0.0391	0.64 (Φ_ST_)	0.0049

Haplotype diversity was higher in the Southeastern group than the Northeast (Table 2). In contrast, nucleotide diversity was equal within the Northeastern and Southeastern groups (Table 2). This indicates that while Northeastern individuals were less likely to have unique haplotypes, the nucleotide differences between individuals, on average, were not smaller than those among Southeastern individuals.

**Table 2:** Genetic diversity among mitochondrially-defined regions (± standard deviation). Diversity is measured as haplotype diversity (*H*), nucleotide diversity (π), and expected heterozygosity (H_e_)

	mtDNA		β-globin	
	*H*	π	H_e_	π
SE group	0.999 ± 0.001	0.009 ± 0.005	0.899 ± 0.017	0.004 ± 0.002
NE group	0.911 ± 0.024	0.009 ± 0.005	0.563 ± 0.053	0.001 ± 0.001
NW group	1.000 ± 0.000	0.004 ± 0.003	0.775 ± 0.068	0.004 ± 0.003
Total	0.988 ± 0.004	0.018 ± 0.006	0.812 ± 0.021	0.003 ± 0.002

### Nuclear genetic variation

18 *N. ocellatus* individuals from 11 localities were initially sequenced for the nuclear genes β-globin and RPS8. Polymorphism at the nucleotide level was much lower for nDNA than mtDNA, representing four characters out of 613 (0.653 %) for RPS8, and 13 out of 656 (1.98 %) for β-globin. Subsequently, β-globin was successfully sequenced for 166 individuals in total (some individuals could not be resolved owing to the presence of heterozygosity for multiple length variants). A total of 55 unique alleles were identified, with 39 (5.8 %) of 670 characters variable, and 30 (4.5 %) parsimony informative.

**Fig. 3:**
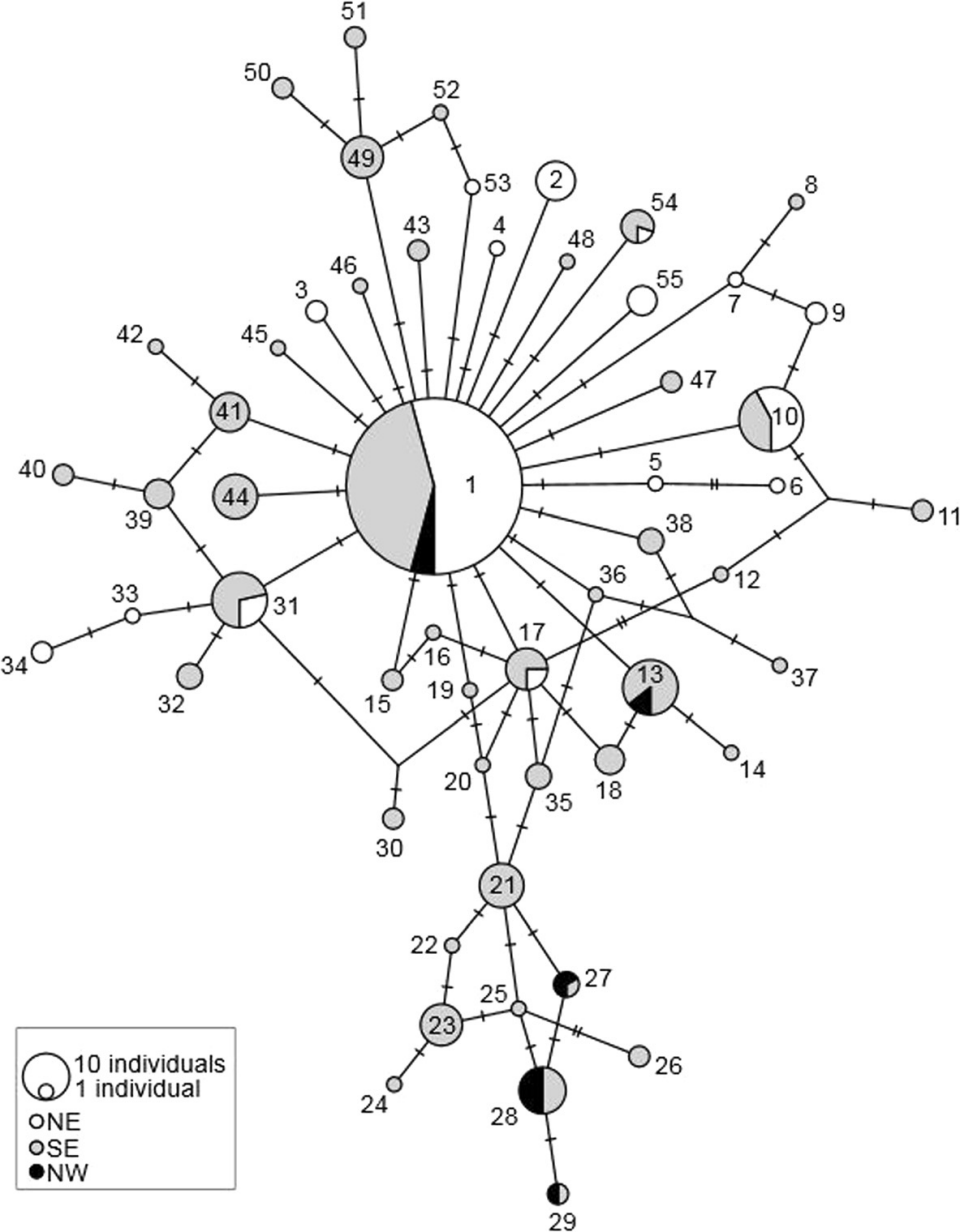
TCS network for nuclear β-globin haplotypes from individuals of *Niveoscincus ocellatus*. Inferred haplotypes are indicated as bars on the links between haplotypes. Fill patterns indicate localities of individuals harbouring haplotypes with respect to the three mitochondrially-defined regions. Numbers correspond to explicit sampling localities of haplotypes as defined in Addtional file 1: Table S3

The TCS network for β-globin revealed limited phylogeographic structure and a lack of consistency with mtDNA relationships (Fig. 3). A common allele was observed in all three mitochondrially-defined regions, at 18 out of 27 localities where nuclear data were obtained. The only suggestion of geographic structuring was for alleles 13–35 (excluding 15, 16, and 31–34), which appear restricted to the western part of the island, and alleles 49–53 which were only observed at Lost Falls, Coles Bay, and Bicheno. During SAMOVA values of Φ_CT_ had plateaued already at two groups, reflecting a western group (Mt Oakleigh, Lake St Clair, Lagoon of Islands, and Strathgordon) and the remainder. There was significant but weak population genetic structuring in the nuclear data when populations were grouped according to the inferred mtDNA groups (Table 1). However, in contrast to mtDNA, β-globin structure among populations, and among populations within groups, was more than an order of magnitude greater than that among groups (Table 1). Also in contrast to mtDNA, there was no suggestion of greater β-globin population genetic structuring in the Northeast than the Southeast. However, heterozygosity was higher in the Southeast than the Northeast, consistent with patterns of mtDNA haplotype diversity (Table 2).

### Demographic histories of regional groups

**Fig. 4:**
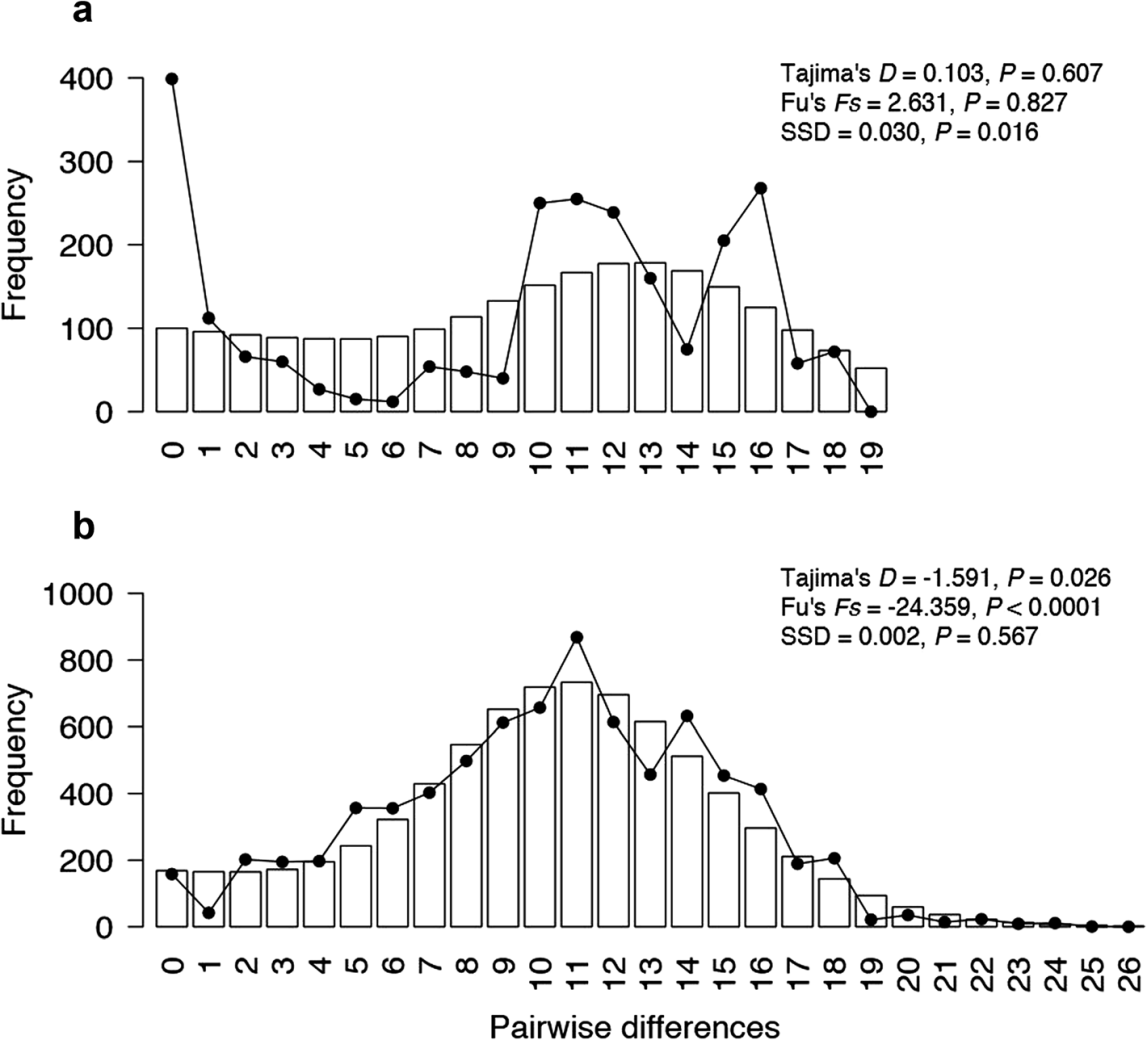
Mitochondrial mismatch distributions for the (**a**) Northeastern and (**b**) Southeastern groups of *Niveoscincus ocellatus*. Histogram reflects coalescent simulation expectations for an exponentially expanding population while the solid line reflects the observed distributions

The Southeastern group was the only group with significant mtDNA Tajima’s *D* and Fu’s *Fs*, and an inability to reject the null hypothesis of population expansion from the mismatch distribution (Fig. 4). The same result was observed when repeating these analyses while excluding Lake Mackenzie from the Southeast group. The Northwest group was not analysed due to the low sample size for this group. Given differences in sample size between the Northeast and Southeast groups (number of individuals per site often large for Northeast sites), we also randomly subsampled these sites to six individuals, but the results were qualitatively identical (non-significant Tajima’s *D* and Fu’s *Fs*, and significant mismatch distribution; haplotype diversity lower, and nucleotide diversity similar to the Southeast group).

**Fig. 5:**
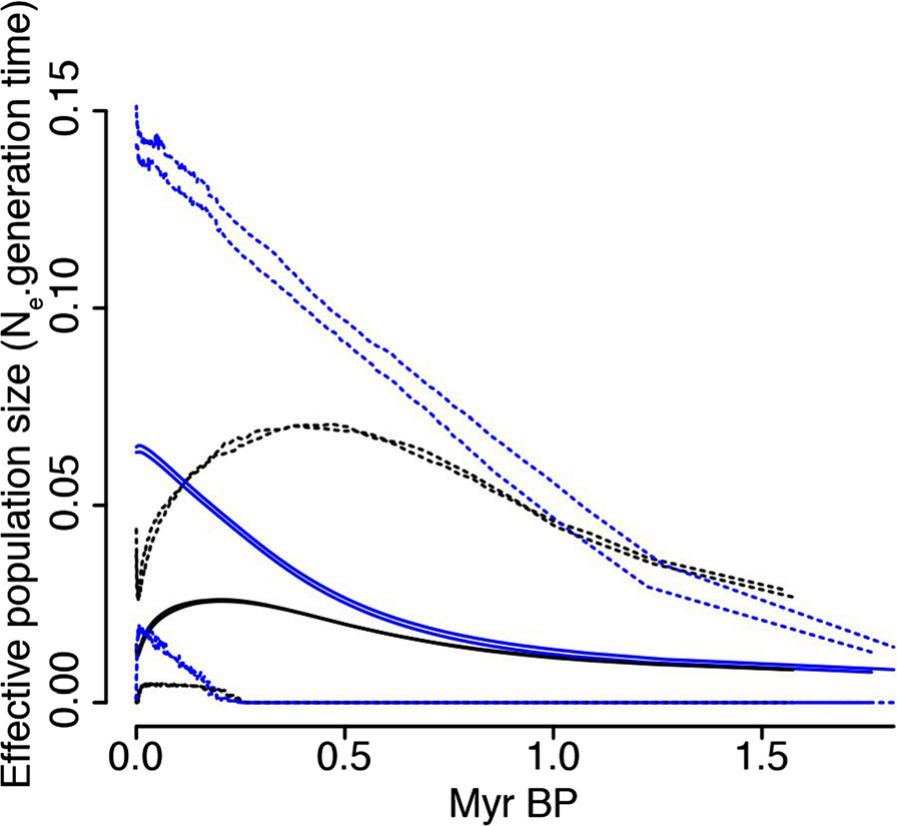
Temporal variation in effective population size of Northeast (black) and Southeast (blue) groups of *Niveoscincus ocellatus*. Solid lines indicate mean, and dashed lines represent 95 % Highest Posterior Densities from replicate Extended Bayesian Skyline Plot analyses

**Fig. 6:**
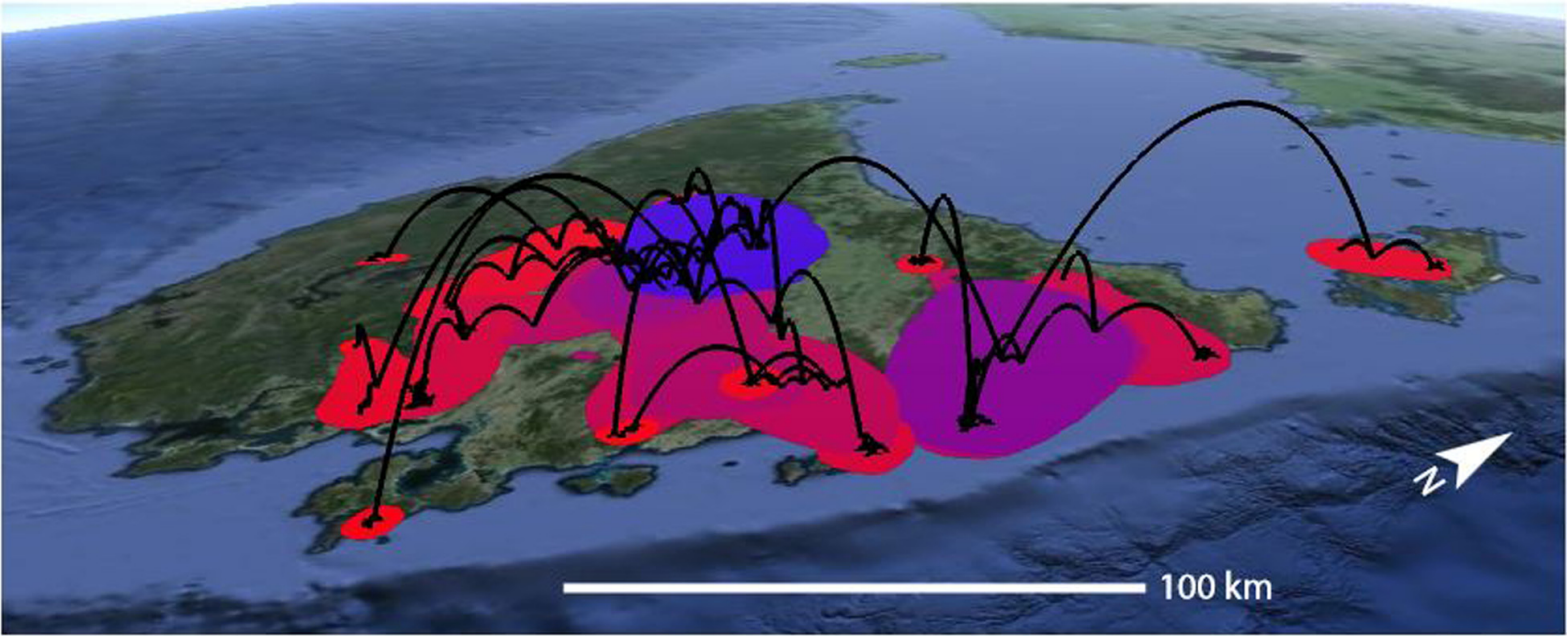
Spatial projection of the relaxed random walk analysis of *Niveoscincus ocellatus*, based on the maximum clade credibility tree (black lines). Coloured areas reflect the 80 % Highest Posterior Density of the distribution of ancestral branches, with dark blue representing the oldest distribution, and red the youngest

Extended Bayesian Skyline Plots conducted on multilocus data also indicated a stronger signal of recent population growth in the Southeast than the Northeast during the last 1.5 Myr, with the Northeast experiencing moderate growth and subsequent decline, and consequently no net change across the plot (Fig. 5). Similarly, the relaxed random walk analysis indicated more frequent and extensive movement in the Southeast than the Northeast (Fig. 6).

## Discussion

Extensive genetic sampling of *N. ocellatus* across its range revealed strong phylogeographic structure at multiple spatial scales. There was broad-scale structure based on mtDNA, with distinct groups corresponding to Tasmania’s Northwest, Northeast and Southeast (the latter extending onto the Central Plateau). Additionally, phylogeographic structuring was evident within Northeast and Southeast groups, although the degree of structuring as well as the demographic history of each region differed. The Northeastern group had the highest levels of internal structure and coalescent analyses did not provide evidence for any substantial net demographic expansion; the populations appear to have persisted in isolation at or near their sampling sites during Pleistocene glaciations without substantial change in population size. This contrasts with the lower spatial structuring and evidence of greater demographic and spatial expansion in the Southeastern group. While populations within the Northwestern group also appeared to have some level of genetic structure, low sampling currently makes it difficult to reliably infer the biogeographic history of populations from this region. The nuclear β-globin locus provided corroborating evidence for the three groups suggested by mtDNA, but only within a population genetic (AMOVA)-rather than phylogeographic—framework. Estimated dates for the divergence of lineages from different regions and demographic expansion were firmly in the Pleistocene, but pre-date the LGM. The observed phylogeographic patterns suggest that Pleistocene climate—but not the LGM specifically-has been highly influential in shaping the distribution of *N. ocellatus* across Tasmania.

### Demographic history

In reptiles, physiological processes essential for survival and reproduction are tightly linked to thermal opportunities [38, 49]. Therefore, the phylogeographic patterns and the demographic histories of *N. ocellatus* populations are likely to reflect aspects of Tasmania’s climatic history. During the Pleistocene, high altitude areas including Ben Lomond, Mt Wellington, Mt Field, and the Tasmanian Central Plateau experienced repeated glacial and periglacial activity [44]. While temperatures in lowland areas also fell during glacial periods, these regions were comparatively warm, experiencing temperatures that were as warm as, or in some cases warmer, than alpine regions inhabited by *N. ocellatus* today [50].

The contrast of phylogeographic patterns in *N. ocellatus* between the highly structured Northeastern group and the comparatively less structured Southeastern group likely reflects historic differences in persistence of *N. ocellatus* populations at or near the sampling sites during the colder, more biologically limiting, glacial periods. The observed diversity of haplotypes and the individually monophyletic status of most sites within the Northeastern group provide support for the climatic suitability of northeast Tasmania for populations of temperate species during or immediately following Pleistocene glaciations, and not just the LGM. Additional support is provided by phylogeographic studies in two plant species, *Eucalyptus regnans* [51] and *Nothofagus cunninghamii* [52], as well as vegetation modelling based on palynological and ecological data, which predicted refugia in this region for rainforest, grassy woodland and eucalypt species during the LGM [50]. In contrast, the non-monophyly of *N. ocellatus* at Ben Lomond is consistent with recolonisation from multiple, low elevation sources.

The monophyletic population structure found in some populations in the Southeastern group including Coles Bay, Fortescue Bay and the combined Russell River and Margate population, indicates that in some of these lowland regions, populations may have either persisted in isolation for longer periods than other Southeast populations, or reflect founder effects from peripheral range expansion, the latter suggested by relaxed random walk analysis (Fig. 6). Regardless, there was a higher level of movement throughout the region presently occupied by the Southeastern group; the genetic structure of populations within this region is comparatively low, with fewer mutations separating individuals from different populations, and with similar or even identical haplotypes shared amongst sites. These phylogeographic patterns are consistent with a history of genetic mixture and demographic instability of populations within the Southeast. Populations likely retreated from temporarily unsuitable locations during glacial periods, becoming conjoined and admixed with other retreating populations or possibly with persisting populations. Range expansion would have occurred when the climate became less severe and more habitats became available, consistent with the observed pattern of demographic and spatial expansion within the Southeastern group. Expansion probably occurred simultaneously from multiple refugial populations, as indicated by the high levels of genetic diversity and non-monophyly of sites such as Lake Mackenzie.

The patterns of glacial response inferred for other Tasmanian taxa bear some similarities with classical patterns in the Northern Hemisphere, where post-glacial recolonisation has often been rapid, and from a single or few source populations at lower latitudes and altitudes, leading to decreases in local genetic diversity [3]. For example, two large lowland refugia in the Storm Bay and Great Oyster Bay (Fig. 1) areas have been inferred for *Eucalyptus globulus* [32]. However, for *N. ocellatus* relaxed random walk analysis suggested persistence or early establishment in Northern Central Tasmania, and Northeast Tasmania immediately thereafter. This bears more similarity to temperate European species that persisted during glacials in ‘cryptic high latitude refugia’, including amphibians and reptiles [53–56]. Furthermore, often what has represented single large refugia in some species (and has contributed to the idea of the classical European patterns) represents multiple glacial refugia for reptiles (e.g., [8, 9, 57, 58]), and may also apply here for *N. ocellatus*.

While molecular clocks are limited in the temporal accuracy they provide [59, 60], estimated dates for lineage divergence and demographic expansion of the Southeastern clade fall well within the Pleistocene period, but predate the LGM (17–20 ka) [25]. The comparatively shallow divergence among the mtDNA clades (*cf.* [17]), as well as the low sequence variability in the nDNA, and lack of congruent phylogeographic structure across mitochondrial and nuclear loci, is also consistent with recent (but pre-LGM) diversification within *N. ocellatus*. The larger effective size of the nDNA markers confers slower lineage sorting relative to mtDNA, which is the favoured explanation for the phylogeographic discordance of these markers (see also [8, 61]).

### Phylogeographic breaks

The location of phylogeographic breaks between the Northwestern, Northeastern and Southeastern clades in *N. ocellatus* do not correspond with any large-scale barriers in the current landscape or match the distribution of any previously reported biogeographic regions [62–64]. However, similar genetic patterns were observed from an RFLP survey of mtDNA variation in *N. metallicus* [65], where significant haplotype frequency differences were evident between the Southeast, Northeast, and Northwest, but sampling distribution, sample sizes, and marker variability in that study were too low for robust comparisons with our patterns. In contrast, there is no evidence of phylogeographic breaks within other Tasmanian reptiles [66–68], and in one instance levels of divergence to mainland conspecifics suggest colonisation after the LGM, but prior to the flooding of Bass Strait [69]; it is important to note that these taxa have more northern distributions than *N. ocellatus*, and are also non-saxicoline, and therefore it might not necessarily be expected that they share phylogeographic histories with *N. ocellatus*.

*Niveoscincus ocellatus* is a habitat specialist with low dispersal capacity [45, 70, 71], and these traits are known to promote population fragmentation, as relatively minor breaks in habitat distribution over short distances can often represent effective barriers to gene flow [72, 73]. If connectivity between populations is low enough it is possible that even geographically close populations may become highly divergent through time. This may account for the divergence between the Coles Bay and Bicheno populations, where although sites are separated by just 30 km, rock coverage is limited and sandy beaches and dense heath dominate this region.

While poor connectivity between populations can explain why dispersal might be rare, it cannot be seen to prevent dispersal completely in *N. ocellatus* given that it currently occupies a wide range of locations not presently connected by rocky habitats. Waters et al. [5] highlight a number of systems where phylogeographic breaks have been linked to density-dependent effects, such as intra-specific competitive exclusion, without current geographic barriers to gene flow. These examples demonstrate that with sufficient levels of intra-specific competition, invaders (at low densities) will be blocked by established (high density) resident populations. This exclusion can operate actively, whereby residents are more likely to win territorial disputes (see Olsson and Shine [74] for an example of this in a congeneric species), restricting invader access to resources and mates. Additionally, this competition can occur at the genetic level, where even if invaders occasionally reproduce successfully, without a selective advantage their genes can be effectively excluded by random genetic drift [5]. There is also evidence for adaptation to local environmental conditions in this species [41, 75, 76], which might further impede gene flow.

Density-dependent effects can maintain barriers to gene flow at a variety of spatial scales [5]. This means that the same processes that maintain the phylogenetic breaks between the major clades of *N. ocellatus* can also explain the maintenance of structure observed within clades such as those in the Northeast, where unique haplotypes are almost always associated with just one population. In addition, because competitive exclusion is density-dependent, it can only explain the maintenance of phylogeographic breaks in demographically stable areas. However, if climatic conditions change and there is an increase in the density of invaders, or an absence of residents in newly available suitable habitat, genes are more likely to move throughout a landscape, and result in a lack of phylogeographic structuring.

## Conclusions

This study indicates that populations of *N. ocellatus* became established and persisted at or near most contemporary sampling locations in Northeast Tasmania, and some low elevation sites in Southeast Tasmania, throughout the LGM and earlier Pleistocene glaciations. Other regions of central and Southeast Tasmania were uninhabited during the Pleistocene glaciations and were not recolonised from a single refugial population. Our findings contribute to the emerging picture of complexity in phylogeographic histories and of responses to Pleistocene glaciations across species and regions globally. Future studies should compare phylogeographic patterns in similar taxa (e.g., *N. metallicus*) to verify the inferences of this study, and also examine the fine-scale spatial distribution of genetic variation between areas identified as Northeast and Southeast clades, to better understand the origin and maintenance of these groups (e.g., [77]).

## Methods

### Sampling

Tissue samples were collected from 204 specimens representing 29 localities (Fig. 1; Additional file 1: Table S1). Sampling was spread across the species’ known latitudinal, longitudinal and altitudinal range (Fig. 1). Field samples included some museum collections (Additional file 1: Table S2) to ensure comprehensive sampling across the species range. *Niveoscincus greeni* and *N. pretiosus* were employed as outgroups.

### DNA extraction, amplification and sequencing

Total genomic DNA was extracted from tail or liver tissue samples following the Qiagen DNeasy Blood and Tissue Extraction Kit (Qiagen, Hilden, Germany). A portion of two mitochondrial genes, ND2 (*c.* 547 bp) and ND4 (*c.* 873 bp), and two nuclear genes, β-globin (exons 2–3, *c.* 656 bp) and RPS8 (40S Ribosomal protein S8 intron 3, *c.* 613 bp), were amplified and sequenced, as they exhibited useful levels of intraspecific variability in previous population studies of squamates (e.g., [17, 78]). The primers used were L4473 and ND2r102 for ND2 [79, 80], ND4l and tRNA-Leu for ND4 [81], LC17 and LC18 for RPS8 [17], and Bglo1CR and Bglo2CR for β-globin [82]. A subset of individuals were initially sequenced in both directions for all fragments, but then only L4437 and tRNA_Leu were employed for subsequent mtDNA sequencing. For the nuclear fragments, only β-globin exhibited sufficient variation for further analysis. Most individuals were sequenced using Bglo1CR, but in instances of heterozygotes for length variation, Bglo2CR was also used for sequencing. PCR reactions were 21 μL and comprised ~20 ng of DNA template, 1 x reaction buffer, 1.9 mM MgCl_2_, 0.14 mM dNTPs, 0.24 mM of each primer, and 0.16 units of *Taq* DNA polymerase (Bioline MangoTaq). For ND2, ND4 and RPS8 amplification was carried out with an initial denaturation at 95 °C for 3 min, followed by 35 cycles of 95 °C denaturation for 25 s, 55 °C annealing for 30 s, and 72 °C extension for 90 s, with a final extension at 72 °C for 5 min after cycling. Amplification for β-globin followed the same protocol but with annealing at 60 °C.

DNA sequence chromatograms were edited and aligned in Geneious version 5.6.4 (Biomatters). Sequence data for ND2 and ND4 were concatenated, providing 1420 bp per individual. TCS v1.21 [83] was used to assign individuals to haplotypes, with attention to ensure that sequences that shared missing data at a given nucleotide position were not assigned to different haplotypes. A single representative of each mitochondrial haplotype was then chosen for phylogeny reconstruction. Given heterozygous nucleotide sites in β-globin, data analyses were conducted on inferred haplotype states rather than using a single sequence with ambiguous nucleotide codes for heterozygous sites. PHASE 2.1.1 [84] was used to phase alleles from heterozygous individuals, with SEQPHASE [85] employed for input and output file processing. DNA sequences analysed are available from GenBank (accession numbers: KJ858058–KJ858494, KP277213–KP277500) and also in Dryad [86].

### Phylogenetic analysis

Maximum parsimony and Bayesian tree-building methods were used for mtDNA data. Maximum parsimony trees were constructed in PAUP* v4.0 [87] using equal weighting of character-state changes, random stepwise sequence addition, and tree-bisection reconnection (TBR) branch swapping. The maximum number of recovered trees was set to 5000, and node support was estimated using 500 bootstrap replicates of the dataset. Bayesian analyses were performed with MrBayes 3.2.2 [88]. Data were partitioned by gene region and analysed under substitution models suggested by JModelTest2 [89, 90] using the Bayesian Information Criterion. MrBayes analyses were performed using the default prior probability distributions for model parameters and duplicate MCMC runs were conducted with four chains of 5,500,000 generations, with a tree sampling frequency of 500 generations. Three of the chains were heated according to ‘Temp = 0.1’. Stationarity and adequate mixing of chains was assessed using Tracer 1.5 [91] to establish the attainment of asymptotes for LnL and substitution model parameters, and to ensure effective sample sizes greater than 200. Convergence was determined when the average standard deviation of split frequencies between runs was lower than 0.02. The first 25 % of the samples were discarded as ‘burn in’ given attainment of stationarity and convergence. Phylogenetic trees were not reconstructed for β-globin given shallow levels of genetic variation. TCS networks were reconstructed for both β-globin and mtDNA using PopART (http://popart.otago.ac.nz/index.shtml).

### Population structuring and demographic histories

Populations were assigned to a regional group based on patterns in the mitochondrial DNA phylogeny and Spatial Analysis of Molecular Variance (SAMOVA; [92]). SAMOVA employed pairwise sequence difference and a range of values for the number of genetic groups, implemented using SAMOVA2 (http://cmpg.unibe.ch/software/samova2/). Population genetic statistics were then calculated for these groups using Arlequin 3.1 [93]. Haplotype diversity and nucleotide diversity were calculated for each group. Population structuring was quantified using Analysis of Molecular Variance (AMOVA) based on haplotype identity. AMOVA calculated hierarchical fixation indices among populations (Φ_ST_), among populations within groups (Φ_SC_), and among groups (Φ_CT_), and P-values were estimated from 1000 permutations of haplotypes. Tajima’s *D* [94] and Fu’s *Fs* [95] were used to test for a signature of demographic expansion for each regional group, assuming selective neutrality of DNA variation. Tests were based on 1000 simulated samples using a coalescent algorithm. Additionally, mismatch distribution analyses were performed to test for exponential demographic expansion [96].

Changes in effective population size were reconstructed using the multilocus Extended Bayesian Skyline Plot (EBSP) [97]. Substitution models were derived from jModelTest2, and a linear model of population size change was implemented. A strict molecular clock was assumed given the shallow levels of divergence, with a mitochondrial mean rate of 1.52 % divergence per million years, with a standard deviation of 0.5 % divergence, based on mtDNA calibrations from other squamates [69]. Analyses were performed using BEAST 1.8.2 [98] with 250 million generations, and sampling every 25,000 generations. Operators were adjusted in accordance with recommendations in the EBSP tutorial at http://beast.bio.ed.ac.uk/tutorials to improve mixing. Replicate runs were tested for convergence visually using Tracer v1.5 [99] and to ensure high effective sample sizes (ESS).

A relaxed random-walk analysis was also employed to reconstruct the spatiotemporal pattern of movements. This approach has similarities to relaxed molecular clock analyses in that it relaxes the constraint of constant movement rates through time [100]. A log-normal prior of movement rates was employed. Analyses were conducted on mtDNA data using BEAST with a coalescent tree prior and strict clock. Locations of individuals were jittered by 0.1 ° latitude and longitude where they were collected at the same site. Replicate analyses of 250 million generations were run to ensure convergence, and a maximum clade credibility tree was constructed using TreeAnnotater v1.8.2 [98]. Visualisation of the spatiotemporal tree was achieved using SPREAD v1.0.6 [101].

### Availability of supporting data

The data set(s) supporting the results of this article are available in the Dryad repository, http://datadryad.org/review?doi=doi:10.5061/dryad.ff32k.

## Additional files

Additional file 1: Table S1. Sampling details of specimens. **Table S2.** Museum specimens. **Table S3.** Sampling locations of haplotypes illustrated in Fig. 3. **Table S4** Sampling locations of haplotypes indicated in Figure S3. **Figure S1.** Maximum parsimony tree for mtDNA variation. **Figure S2.** Maximum clade credibility tree from BEAST analysis. **Figure S3.** Minimum spanning network for mtDNA variation.

## References

[1] Hewitt G (2000). The genetic legacy of the Quaternary ice ages. Nature.

[2] Provan J, Bennett KD (2008). Phylogeographic insights into cryptic glacial refugia. Trends Ecol Evol.

[3] Hewitt GM (2004). Genetic consequences of climatic oscillations in the Quaternary. Philos Trans R Soc Lond B Biol Sci.

[4] Taberlet P, Fumagalli L, Wust-Saucy A-G, Cosson J-F (1998). Comparative phylogeography and postglacial colonization routes in Europe. Mol Ecol.

[5] Waters JM, Fraser CI, Hewitt GM (2013). Founder takes all: density-dependent processes structure biodiversity. Trends Ecol Evol.

[6] Stewart JR, Lister AM, Barnes I, Dalén L (2010). Refugia revisited: individualistic responses of species in space and time. Proc R Soc B Biol Sci.

[7] Schmitt T, Varga Z (2012). Extra-Mediterranean refugia: the rule and not the exception. Fron t Zool.

[8] Salvi D, Harris DJ, Kaliontzopoulou A, Carretero MA, Pinho C (2013). Persistence across Pleistocene ice ages in Mediterranean and extra-Mediterranean refugia: phylogeographic insights from the common wall lizard. BMC Evol Biol.

[9] Gómez A, Lunt DH: Refugia within refugia: patterns of phylogeographic concordance in the Iberian Peninsula. In: Phylogeography of southern European refugia. Dordrecht, The Netherlands: Springer; 2007: 155–188.

[10] Beheregaray LB (2008). Twenty years of phylogeography: the state of the field and the challenges for the Southern Hemisphere. Mol Ecol.

[11] Dubey S, Shine R (2011). Geographic variation in the age of temperate-zone reptile and amphibian species: Southern Hemisphere species are older. Biol Lett.

[12] Dubey S, Shine R (2012). Are reptile and amphibian species younger in the Northern Hemisphere than in the Southern Hemisphere?. J Evol Biol.

[13] Tingley R, Dubey S. Disparity in the timing of vertebrate diversification events between the northern and southern hemispheres. BMC Evol Biol. 2012;12. 10.1186/1471-2148-12-244 PMC3540028 23241454

[14] Turchetto-Zolet AC, Pinheiro F, Salgueiro F, Palma-Silva C (2013). Phylogeographical patterns shed light on evolutionary process in South America. Mol Ecol.

[15] Fraser CI, Nikula R, Ruzzante DE, Waters JM (2012). Poleward bound: biological impacts of Southern Hemisphere glaciation. Trends Ecol Evol.

[16] Costa LP (2003). The historical bridge between the Amazon and the Atlantic Forest of Brazil: a study of molecular phylogeography with small mammals. J Biogeogr.

[17] Bell RC, Parra JL, Tonione M, Hoskin CJ, Mackenzie JB, Williams SE, Moritz C (2010). Patterns of persistence and isolation indicate resilience to climate change in montane rainforest lizards. Mol Ecol.

[18] Hoskin CJ, Tonione M, Higgie M, MacKenzie JB, Williams SE, VanDerWal J, Moritz C (2011). Persistence in peripheral refugia promotes phenotypic divergence and speciation in a rainforest frog. Am Nat.

[19] Barlow A, Baker K, Hendry CR, Peppin L, Phelps T, Tolley KA, Wüster CE, Wüster W (2013). Phylogeography of the widespread African puff adder (Bitis arietans) reveals multiple Pleistocene refugia in southern Africa. Mol Ecol.

[20] Ritchie PA, Millar CD, Gibb GC, Baroni C, Lambert DM (2004). Ancient DNA enables timing of the Pleistocene origin and Holocene expansion of two Adélie penguin lineages in Antarctica. Mol Biol Evol.

[21] Fraser CI, Nikula R, Spencer HG, Waters JM (2009). Kelp genes reveal effects of subantarctic sea ice during the Last Glacial Maximum. Proc Natl Acad Sci.

[22] Wallis GP, Trewick SA (2009). New Zealand phylogeography: evolution on a small continent. Mol Ecol.

[23] McGaughran A, Torricelli G, Carapelli A, Frati F, Stevens MI, Convey P, Hogg ID (2010). Contrasting phylogeographical patterns for springtails reflect different evolutionary histories between the Antarctic Peninsula and continental Antarctica. J Biogeogr.

[24] Sersic AN, Cosacov A, Cocucci AA, Johnson LA, Pozner R, Avila LJ, SITES J, Morando M (2011). Emerging phylogeographical patterns of plants and terrestrial vertebrates from Patagonia. Biol J Linn Soc.

[25] Barrows TT, Stone JO, Fifield LK, Cresswell RG (2002). The timing of the last glacial maximum in Australia. Quat Sci Rev.

[26] Colhoun EA (1985). Glaciations of the west coast range. Tasmania. Quatern Res.

[27] Kiernan K. The extent of late Cenozoic glaciation in the Central Highlands of Tasmania, Australia. Arct Alp Res. 1990;341–354.

[28] Colhoun EA, Hannan D, Kiernan K (1996). Late Wisconsin glaciation of Tasmania. Papers and proceedings of the royal society of Tasmania.

[29] Augustinus PC, Macphail MK (1997). Early Pleistocene stratigraphy and timing of the Bulgobac Glaciation, western Tasmania, Australia. Palaeogeogr Palaeoclimatol Palaeoecol.

[30] Kiernan K, Lauritzen SE, Duhig N (2001). Glaciation and cave sediment aggradation around the margins of the Mt Field Plateau, Tasmania. Aust J Earth Sci.

[31] Mackintosh A, Barrows T, Colhoun E, Fifield L (2006). Exposure dating and glacial reconstruction at Mt. Field, Tasmania, Australia, identifies MIS 3 and MIS 2 glacial advances and climatic variability. J Quat Sci.

[32] McKinnon GE, Jordan GJ, Vaillancourt RE, Steane DA, Potts BM (2004). Glacial refugia and reticulate evolution: the case of the Tasmanian eucalypts. Philos Trans R Soc Lond B Biol Sci.

[33] Byrne M (2008). Evidence for multiple refugia at different time scales during Pleistocene climatic oscillations in southern Australia inferred from phylogeography. Quat Sci Rev.

[34] Rull V (2009). Microrefugia. J Biogeogr.

[35] Lessa EP, D’Elia G, Pardinas UFJ (2010). Genetic footprints of late Quaternary climate change in the diversity of Patagonian-Fueguian rodents. Mol Ecol.

[36] Zhang Z-Y, Cashins S, Philips A, Burridge CP (2014). Significant population genetic structuring but a lack of phylogeographic structuring in the endemic Tasmanian tree frog (*Litoria burrowsae*). Aust J Zool.

[37] Camargo A, Sinervo B, Sites JW (2010). Lizards as model organisms for linking phylogeographic and speciation studies. Mol Ecol.

[38] Angilletta MJ, Niewiarowski PH, Navas CA (2002). The evolution of thermal physiology in ectotherms. J Therm Biol.

[39] Araújo MB, Nogués-Bravo D, Diniz-Filho JAF, Haywood AM, Valdes PJ, Rahbek C (2008). Quaternary climate changes explain diversity among reptiles and amphibians. Ecography.

[40] Rawlinson P: Biogeography and ecology of the reptiles of Tasmania and the Bass Strait area. In: Biogeography and ecology in Tasmania. Springer; 1974: 291–338

[41] Uller T, While GM, Cadby CD, Harts A, O’Connor K, Pen I, Wapstra E (2011). Altitudinal divergence in maternal thermoregulatory behaviour may be driven by differences in selection on offspring survival in a viviparous lizard. Evolution.

[42] Wapstra E, Swain R. Geographic and annual variation in life-history traits in a temperate zone Australian skink. J Herpetol. 2001;194–203.

[43] Olsson M, Shine R, Wapstra E, Ujvari B, Madsen T (2002). Sexual dimorphism in lizard body shape: the roles of sexual selection and fecundity selection. Evolution.

[44] Colhoun EA (2002). Periglacial landforms and deposits of Tasmania: Periglacial and Permafrost Research in the Southern Hemisphere. S Afr J Sci.

[45] Melville J (2007). Evolutionary correlations between microhabitat specialisation and locomotor capabilities in the lizard genus Niveoscincus. Aust J Zool.

[46] Gardner MG, Godfrey SS, Fenner AL, Donnellan SC, Bull CM (2012). Fine-scale spatial structuring as an inbreeding avoidance mechanism in the social skink Egernia stokesii. Aust J Zool.

[47] Berry O, Tocher MD, Sarre SD (2004). Can assignment tests measure dispersal?. Mol Ecol.

[48] Dennison S, Smith SM, Stow AJ (2012). Long-distance geneflow and habitat specificity of the rock-dwelling coppertail skink, Ctenotus taeniolatus. Austral Ecol.

[49] Sinervo B, Mendez-De-La-Cruz F, Miles DB, Heulin B, Bastiaans E, Villagrán-Santa Cruz M, Lara-Resendiz R, Martínez-Méndez N, Calderón-Espinosa ML, Meza-Lázaro RN (2010). Erosion of lizard diversity by climate change and altered thermal niches. Science.

[50] Kirkpatrick JB, Fowler M (1998). Locating likely glacial forest refugia in Tasmania using palynological and ecological information to test alternative climatic models. Biol Conserv.

[51] Nevill PG, Bossinger G, Ades PK (2010). Phylogeography of the world’s tallest angiosperm, Eucalyptus regnans: evidence for multiple isolated Quaternary refugia. J Biogeogr.

[52] Worth JR, Jordan GJ, McKinnon GE, Vaillancourt RE (2009). The major Australian cool temperate rainforest tree Nothofagus cunninghamii withstood Pleistocene glacial aridity within multiple regions: evidence from the chloroplast. New Phytol.

[53] Ursenbacher S, Carlsson M, Helfer V, Tegelström H, Fumagalli L (2006). Phylogeography and Pleistocene refugia of the adder (Vipera berus) as inferred from mitochondrial DNA sequence data. Mol Ecol.

[54] Ursenbacher S, Conelli A, Golay P, Monney J-C, Zuffi M, Thiery G, Durand T, Fumagalli L (2006). Phylogeography of the asp viper (*Vipera aspis*) inferred from mitochondrial DNA sequence data: Evidence for multiple Mediterranean refugial areas. Mol Phylogenet Evol.

[55] Hofman S, Spolsky C, Uzzell T, COGĂLNICEANU D, BABIK W, Szymura JM (2007). Phylogeography of the fire‐bellied toads Bombina: independent Pleistocene histories inferred from mitochondrial genomes. Mol Ecol.

[56] Teacher AGF, Garner TWJ, Nichols RA (2009). European phylogeography of the common frog (Rana temporaria): routes of postglacial colonization into the British Isles, and evidence for an Irish glacial refugium. Heredity.

[57] Podnar M, Mayer W, Tvrtković N (2005). Phylogeography of the Italian wall lizard, Podarcis sicula, as revealed by mitochondrial DNA sequences. Mol Ecol.

[58] Canestrelli D, Cimmaruta R, Nascetti G (2008). Population genetic structure and diversity of the Apennine endemic stream frog, Rana italica–insights on the Pleistocene evolutionary history of the Italian peninsular biota. Mol Ecol.

[59] Bromham L, Penny D (2003). The modern molecular clock. Nat Rev Genet.

[60] Ho SYW, Larson G (2006). Molecular clocks: when times are a-changin’. Trends Genet.

[61] Miraldo A, Hewitt GM, Paulo OS, Emerson BC (2011). Phylogeography and demographic history of Lacerta lepida in the Iberian Peninsula: multiple refugia, range expansions and secondary contact zones. BMC Evol Biol.

[62] Shiel R, Koste W, Tan L: Tasmania revisited: rotifer communities and habitat heterogeneity. In: Rotifer Symposium V: 1989. Springer: 239–245.

[63] Mesibov R (1994). Faunal breaks in Tasmania and their significance for invertebrate conservation. Mem Queensland Mus.

[64] Vélez S, Mesibov R, Giribet G (2012). Biogeography in a continental island: population structure of the relict endemic centipede Craterostigmus tasmanianus (Chilopoda, Craterostigmomorpha) in Tasmania using 16S rRNA and COI. J Hered.

[65] McCoull CJ: Geographic variation and adaptation in the Tasmanian metallic skink (*Niveoscincus metallicus*) PhD thesis, University of Tasmania; 2001.

[66] Keogh JS, Scott IA, Hayes C (2005). Rapid and repeated origin of insular gigantism and dwarfism in Australian tiger snakes. Evolution.

[67] Dubey S, Shine R (2010). Evolutionary diversification of the lizard genus *Bassiana* (Scincidae) across Southern Australia. PLoS ONE.

[68] Chapple DG, Hoskin CJ, Chapple SNJ, Thompson MB. Phylogeographic divergence in the widespread delicate skink (Lampropholis delicata) corresponds to dry habitat barriers in eastern Australia. BMC Evol Biol. 2011;11. 10.1186/1471-2148-11-191 PMC3141439 21726459

[69] Chapple DG, Chapple SN, Thompson MB (2011). Biogeographic barriers in south-eastern Australia drive phylogeographic divergence in the garden skink, Lampropholis guichenoti. J Biogeogr.

[70] Atkins N, Swain R, Wapstra E, Jones SM (2007). Late stage deferral of parturition in the viviparous lizard Niveoscincus ocellatus (Gray 1845): implications for offspring quality and survival. Biol J Linn Soc.

[71] Wapstra E, Uller T, While G, Olsson M, Shine R (2010). Giving offspring a head start in life: field and experimental evidence for selection on maternal basking behaviour in lizards. J Evol Biol.

[72] Henle K, Lindenmayer DB, Margules CR, Saunders DA, Wissel C (2004). Species survival in fragmented landscapes: where are we now?. Biodivers Conserv.

[73] Ayre DJ, Minchinton TE, Perrin C (2009). Does life history predict past and current connectivity for rocky intertidal invertebrates across a marine biogeographic barrier?. Mol Ecol.

[74] Olsson M, Shine R (2000). Ownership influences the outcome of male-male contests in the scincid lizard, Niveoscincus microlepidotus. Behav Ecol.

[75] Pen I, Uller T, Feldmeyer B, Harts A, While GM, Wapstra E (2010). Climate-driven population divergence in sex-determining systems. Nature.

[76] Cadby CD, Jones SM, Wapstra E (2014). Geographical differences in maternal basking behaviour and offspring growth rate in a climatically widespread viviparous reptile. J Exp Biol.

[77] Phillips BL, Baird SJ, Moritz C (2004). When vicars meet: a narrow contact zone between morphologically cryptic phylogeographic lineages of the rainforest skink, Carlia rubrigularis. Evol.

[78] Greaves SN, Chapple DG, Gleeson DM, Daugherty CH, Ritchie PA (2007). Phylogeography of the spotted skink (*Oligosoma lineoocellatum*) and green skink (*O. chloronoton*) species complex (Lacertilia: Scincidae) in New Zealand reveals pre-Pleistocene divergence. Mol Phylogenet Evol.

[79] Macey JR, Larson A, Ananjeva NB, Fang Z, Papenfuss TJ (1997). Two novel gene orders and the role of light-strand replication in rearrangement of the vertebrate mitochondrial genome. Mol Biol Evol.

[80] Sadlier RA, Smith SA, Bauer AM, Whitaker AH (2004). A new genus and species of live-bearing scincid lizard (Reptilia: Scincidae) from New Caledonia. J Herpetol.

[81] Forstner MRJ, Davis SK, Arévalo E (1995). Support for the hypothesis of anguimorph ancestry for the suborder Serpentes from phylogenetic analysis of mitochondrial DNA sequences. Mol Phylogenet Evol.

[82] Dolman G, Phillips B (2004). Single copy nuclear DNA markers characterized for comparative phylogeography in Australian wet tropics rainforest skinks. Mol Ecol Notes.

[83] Clement M, Posada D, Crandall KA (2000). TCS: a computer program to estimate gene genealogies. Mol Ecol.

[84] Stephens M, Smith NJ, Donnelly P (2001). A new statistical method for haplotype reconstruction from population data. Am J Hum Genet.

[85] Flot JF (2010). SeqPHASE: a web tool for interconverting PHASE input/output files and FASTA sequence alignments. Mol Ecol Res.

[86] Cliff HC, Wapstra E, Burridge CP: Data from: Persistence and dispersal in a Southern Hemisphere glaciated landscape: the phylogeography of the spotted snow skink (*Niveoscincus ocellatus*) in Tasmania. Dryad. 2015. http://datadryad.org/review?doi=doi:10.5061/dryad.ff32k. 10.1186/s12862-015-0397-y PMC4482293 26111715

[87] Swofford DL (2003). PAUP*. Phylogenetic analysis using parsimony (* and other methods). Version 4.

[88] Ronquist F, Huelsenbeck JP (2003). MrBayes 3: Bayesian phylogenetic inference under mixed models. Bioinformatics.

[89] Darriba D, Taboada GL, Doallo R, Posada D (2012). jModelTest 2: more models, new heuristics and parallel computing. Nat Methods.

[90] Guindon S, Gascuel O (2003). A simple, fast, and accurate algorithm to estimate large phylogenies by maximum likelihood. Syst Biol.

[91] Rambaut A, Suchard MA, Xie D, Drummond AJ: Tracer v1.5. In.: Available from http://beast.bio.ed.ac.uk/Tracer; 2013.

[92] Dupanloup I, Schneider S, Excoffier L (2002). A simulated annealing approach to define the genetic structure of populations. Mol Ecol.

[93] Excoffier L, Laval G, Schneider S (2005). Arlequin (version 3.0): an integrated software package for population genetics data analysis. Evol Bioinformatics Online.

[94] Tajima F (1989). The effect of change in population size on DNA polymorphism. Genet.

[95] Fu Y-X (1997). Statistical tests of neutrality of mutations against population growth, hitchhiking and background selection. Genet.

[96] Rogers AR, Harpending H (1992). Population growth makes waves in the distribution of pairwise genetic differences. Mol Biol Evol.

[97] Heled J, Drummond AJ. Bayesian inference of population size history from multiple loci. BMC Evol Biol. 2008;8. 10.1186/1471-2148-8-289 PMC2636790 18947398

[98] Drummond AJ, Suchard MA, Xie D, Rambaut A (2012). Bayesian Phylogenetics with BEAUti and the BEAST 1.7. Mol Biol Evol.

[99] Rambaut A, Drummond A: Tracer v1. 4. In.: Available from http://beast.bio.ed.ac.uk/Tracer; 2007.

[100] Lemey P, Rambaut A, Welch JJ, Suchard MA (2010). Phylogeography takes a relaxed random walk in continuous space and time. Mol Biol Evol.

[101] Bielejec F, Rambaut A, Suchard MA, Lemey P (2011). SPREAD: spatial phylogenetic reconstruction of evolutionary dynamics. Bioinformatics.

