# Supplementary material for: Persistence and dispersal in a Southern Hemisphere glaciated landscape: the phylogeography of the spotted snow skink (Niveoscincus ocellatus) in Tasmania
Source: BMC Evol Biol. 2015 Jun 26;15:121. doi: 10.1186/s12862-015-0397-y (PMC4482293; doi:10.1186/s12862-015-0397-y)
Supplement: Supplementary file 1 — Sampling details of specimens. Table S2. Museum specimens. Table S3. Sampling locations of haplotypes illustrated in Fig. 3. Table S4 Sampling locations of haplotypes indicated in Figure S3. Figure S1. Maximum parsimony tree for mtDNA variation. Figure S2. Maximum clade credibility tree from BEAST analysis. Figure S3. Minimum spanning network for mtDNA variation. [file 12862_2015_397_MOESM1_ESM.docx]

**Table S1.** Locality coordinates for each of the 29 *Niveoscincus ocellatus* populations used in this study. *localities where DNA samples were obtained from museum specimens.

| \| Clade \| Locality \| Latitude °S \| Longitude °E \| mtDNA samples (n) \| nDNA samples (n) \| \| --- \| --- \| --- \| --- \| --- \| --- \| \| Northeast \| Ben Lomond \| -41.5085 \| 147.6370 \| 6 \| 5 \| \|  \| Bicheno \| -41.8759 \| 148.3108 \| 18 \| 17 \| \|  \| Cataract Gorge \| -41.4595 \| 147.1116 \| 20 \| 15 \| \|  \| Scottsdale \| -41.1630 \| 147.5622 \| 1 \| 1 \| \|  \| Coles Bay \| -42.1223 \| 148.3419 \| 20 \| 18 \| \|  \| Flinders Island* \| -40.2375 \| 148.1121 \| 4 \| 3 \| \|  \| Goose Island* \| -40.2992 \| 147.7958 \| 2 \| - \| \|  \| The Gardens \| -41.1623 \| 148.2838 \| 19 \| 18 \| \|  \|  \|  \|  \|  \|  \| \| Southeast \| Bushy Park \| -42.6787 \| 146.7549 \| 5 \| 5 \| \|  \| Fortescue Bay \| -43.1383 \| 147.9567 \| 6 \| 5 \| \|  \| Halfmoon Creek \| -41.7499 \| 146.7122 \| 7 \| 5 \| \|  \| Lagoon of Islands \| -42.1110 \| 146.9355 \| 4 \| 4 \| \|  \| Lake Echo \| -42.2167 \| 146.6387 \| 3 \| 3 \| \|  \| Lake Mackenzie \| -41.6801 \| 146.3828 \| 5 \| 3 \| \|  \| Lake St Clair \| -42.1152 \| 146.1794 \| 6 \| 5 \| \|  \| Lost Falls \| -42.0431 \| 147.8915 \| 12 \| 8 \| \|  \| Margate \| -43.0210 \| 147.2649 \| 6 \| 4 \| \|  \| Miena \| -41.9811 \| 146.7301 \| 6 \| 9 \| \|  \| Mt Field \| -42.6761 \| 146.6255 \| 6 \| 5 \| \|  \| Mt Nelson \| -42.9236 \| 147.3439 \| 7 \| 4 \| \|  \| Mt Wellington \| -42.8897 \| 147.2356 \| 6 \| 5 \| \|  \| Orford \| -42.5563 \| 147.8321 \| 7 \| 6 \| \|  \| Ridgeway \| -42.9105 \| 147.2964 \| 3 \| - \| \|  \| Ross* \| -42.0278 \| 147.3220 \| 2 \| 2 \| \|  \| Russell River \| -42.9414 \| 146.7880 \| 2 \| 2 \| \|  \| Strathgordon \| -42.7366 \| 145.9781 \| 5 \| 3 \| \|  \| Tooms Lake \| -42.2143 \| 147.7529 \| 8 \| 5 \| \|  \|  \|  \|  \|  \|  \| \| Northwest \| Dove Lake* \| -41.6512 \| 145.9615 \| 3 \| 3 \| \|  \| Mt Oakleigh* \| -41.8056 \| 146.0374 \| 5 \| 5 \| \|  \| **Total samples** \|  \|  \| **204** \| **168** \| |
| --- | --- | --- | --- | --- | --- | --- | --- | --- | --- | --- | --- | --- | --- | --- | --- | --- | --- | --- | --- | --- | --- | --- | --- | --- | --- | --- | --- | --- | --- | --- | --- | --- | --- | --- | --- | --- | --- | --- | --- | --- | --- | --- | --- | --- | --- | --- | --- | --- | --- | --- | --- | --- | --- | --- | --- | --- | --- | --- | --- | --- | --- | --- | --- | --- | --- | --- | --- | --- | --- | --- | --- | --- | --- | --- | --- | --- | --- | --- | --- | --- | --- | --- | --- | --- | --- | --- | --- | --- | --- | --- | --- | --- | --- | --- | --- | --- | --- | --- | --- | --- | --- | --- | --- | --- | --- | --- | --- | --- | --- | --- | --- | --- | --- | --- | --- | --- | --- | --- | --- | --- | --- | --- | --- | --- | --- | --- | --- | --- | --- | --- | --- | --- | --- | --- | --- | --- | --- | --- | --- | --- | --- | --- | --- | --- | --- | --- | --- | --- | --- | --- | --- | --- | --- | --- | --- | --- | --- | --- | --- | --- | --- | --- | --- | --- | --- | --- | --- | --- | --- | --- | --- | --- | --- | --- | --- | --- | --- | --- | --- | --- | --- | --- | --- | --- | --- | --- | --- | --- | --- | --- | --- | --- | --- | --- | --- | --- | --- | --- |

**Table S2.** Details of museum samples used in genetic analyses. Samples are from the Australian National Wildlife Collection (ANWC), the South Australian Museum (SAM) and the Queen Victoria Museum and Art Gallery (QVMAG).

| Population | Museum | Catalogue Number |
| --- | --- | --- |
| Ross | ANWC | R05625 |
| Ross | ANWC | R05628 |
| Mt Oakleigh | SAM | TMHC874 (ABTC23118) |
| Mt Oakleigh | SAM | TMHC876 (ABTC23120) |
| Mt Oakleigh | SAM | TMHC877 (ABTC23121) |
| Mt Oakleigh | SAM | TMHC878 (ABTC23122) |
| Mt Oakleigh | SAM | TMHC879 (ABTC23123) |
| Flinders Island | SAM | NMVD62264 (ABTC23564) |
| Flinders Island | SAM | NMVD62263 (ABTC23563) |
| Flinders Island | SAM | NMVD62262 (ABTC23562) |
| Flinders Island | SAM | NMVD62261 (ABTC23561) |
| Dove Lake | SAM | TMHC485 (ABTC23227) |
| Dove Lake | SAM | TMHC486 (ABTC23228) |
| Dove Lake | SAM | TMHC460 (ABTC23201) |
| Goose Island | QVMAG | QVM:1986:3:0067 |
| Goose Island | QVMAG | QVM:1986:3:0085 |

**Table S3.** Frequency and distribution of β-globin alleles in *Niveoscincus ocellatus* as indicated in Figure 4.

| Allele# (Fig. 4) | Allele sampling locations (numbers in parentheses indicate frequencies when greater than one) |
| --- | --- |
| 1 | Bicheno (33), Coles Bay (27), Cataract Gorge (9), Dove Lake (4), Fortescue Bay, Flinders Is. (6), Gardens (32), Lost Falls (5), Lake Mackenzie, Mt. Field, Margate, Miena (2), Mt. Nelson (2), Mt. Oakleigh (2), Orford (4), Halfmoon Creek (3), Tooms Lake (6), Mt Wellington (3) |
| 2 | Bicheno (7) |
| 3 | Scottsdale (2) |
| 4 | Bicheno |
| 5 | Cataract Gorge |
| 6 | Cataract Gorge |
| 7 | Bicheno |
| 8 | Lake Echo |
| 9 | Cataract Gorge (2) |
| 10 | Bicheno, Ben Lomond (2), Bushy Park, Cataract Gorge (8), Lost Falls, Mt. Field (3), Orford (3) |
| 11 | Orford (2) |
| 12 | Halfmoon Creek |
| 13 | Bushy Park (3), Lake St Clair (2), Dove Lake (2), Lake Echo, Lake Mackenzie (2), Mt. Field (2), Margate, Halfmoon Creek |
| 14 | Lake St. Clair |
| 15 | Orford, Mt. Wellington |
| 16 | Mt. Wellington |
| 17 | Cataract Gorge (2), Lake Echo, Lagoon of Islands, Lake Mackenzie, Miena(2), Russell River |
| 18 | Lake Echo, Lagoon of Islands, Lake Mackenzie, Mt. Field |
| 19 | Halfmoon Creek |
| 20 | Mt. Wellington |
| 21 | Lagoon of Islands (4), Miena, Strathgordon (4) |
| 22 | Lake St. Clair |
| 23 | Bushy Park, Lake St Clair (6), Miena |
| 24 | Miena |
| 25 | Lagoon of Islands |
| 26 | Halfmoon Creek (2) |
| 27 | Lake Mackenzie, Mt. Oakleigh (2) |
| 28 | Lake Echo, Lagoon of Islands, Miena (3), Mt Oakleigh (5) |
| 29 | Miena, Mt Oakleigh |
| 30 | Mt. Wellington (2) |
| 31 | Cataract Gorge (4), Lost Falls, Margate (2), Mt. Nelson (3), Russell River (3), Mt. Wellington |
| 32 | Mt. Nelson (2), Mt. Wellington |
| 33 | Cataract Gorge |
| 34 | Cataract Gorge (2) |
| 35 | Miena, Strathgordon (2) |
| 36 | Tooms Lake |
| 37 | Mt. Nelson |
| 38 | Orford (2), Halfmoon Creek |
| 39 | Bushy Park, Mt. Field (3) |
| 40 | Lake Echo, Lost Falls |
| 41 | Bushy Park, Ross, Lost Falls, Margate (4) |
| 42 | Tooms Lake |
| 43 | Lake Echo (2) |
| 44 | Fortescue Bay (9) |
| 45 | Lost Falls |
| 46 | Halfmoon Creek |
| 47 | Tooms Lake (2) |
| 48 | Lake Echo |
| 49 | Coles Bay (7), Lost Falls |
| 50 | Coles Bay (2) |
| 51 | Coles Bay (2) |
| 52 | Lost Falls |
| 53 | Bicheno |
| 54 | Bicheno, Lost Falls (4) |
| 55 | Gardens (4) |

**Table S4.** Frequency and distribution of mitochondrial haplotypes in *Niveoscincus ocellatus* as indicated in Figure S2 (restricted to sequences with no missing data at known polymorphic sites).

| Haplotype# (Fig. S1) | Haplotype sampling locations (numbers in parentheses indicate frequencies when greater than one) |
| --- | --- |
| 1 | Gardens (3) |
| 2 | Gardens (10) |
| 3 | Gardens |
| 4 | Gardens |
| 5 | Gardens (2) |
| 6 | Gardens (3) |
| 7 | Scottsdale |
| 8 | Bicheno (16) |
| 9 | Ben Lomond |
| 10 | Ben Lomond |
| 11 | Ben Lomond (2) |
| 12 | Ben Lomond (2) |
| 13 | Cataract Gorge (4) |
| 14 | Cataract Gorge (2) |
| 15 | Cataract Gorge |
| 16 | Cataract Gorge (3) |
| 17 | Cataract Gorge |
| 18 | Cataract Gorge (2) |
| 19 | Cataract Gorge |
| 20 | Lake St. Clair (2), Lake Echo |
| 21 | Tooms Lake |
| 22 | Tooms Lake |
| 23 | Lake Echo |
| 24 | Lake Echo |
| 25 | Tooms Lake |
| 26 | Lagoon of Islands |
| 27 | Lagoon of Islands |
| 28 | Lake Echo (2) |
| 29 | Mt. Field |
| 30 | Lake St. Clair |
| 31 | Strathgordon (5) |
| 32 | Mt. Field |
| 33 | Mt. Field |
| 34 | Mt. Field (3) |
| 35 | Mt. Wellington (3) |
| 36 | Lake Mackenzie |
| 37 | Mt. Nelson (3) |
| 38 | Mt. Wellington (2) |
| 39 | Fortescue Bay (2) |
| 40 | Fortescue Bay |
| 41 | Fortescue Bay (3) |
| 42 | Orford |
| 43 | Mt. Nelson (4) |
| 44 | Halfmoon Creek |
| 45 | Halfmoon Creek |
| 46 | Lost Falls (2) |
| 47 | Lost Falls |
| 48 | Orford (2) |
| 49 | Coles Bay (5) |
| 50 | Orford |
| 51 | Lost Falls |
| 52 | Lost Falls (2) |
| 53 | Lost Falls |
| 54 | Tooms Lake |
| 55 | Halfmoon Creek |
| 56 | Halfmoon Creek |
| 57 | Lake Mackenzie |
| 58 | Lake Mackenzie |
| 59 | Halfmoon Creek |
| 60 | Lagoon of Islands |
| 61 | Lagoon of Islands |
| 62 | Bushy Park (3) |
| 63 | Margate |
| 64 | Margate (2) |
| 65 | Russell River (2) |
| 66 | Margate |
| 67 | Miena |
| 68 | Miena |
| 69 | Miena |
| 70 | Halfmoon Creek |
| 71 | Miena |
| 72 | Mt. Oakleigh |
| 73 | Coles Bay (10) |
| 74 | Coles Bay |
| 75 | Coles Bay |
| 76 | Ridgeway |
| 77 | Ridgeway |
| 78 | Mt. Wellington |

**Figure S1.** A minimum length tree (456 steps) from maximum parsimony analysis based on 1420 bp of ND2 and ND4 DNA sequence from *Niveoscincus ocellatus*. Branch lengths are scaled proportional to the number of steps. Numbers in parentheses indicate the number of individuals from a site exhibiting that haplotype. Grey boxes highlight regionally monophyletic areas within the major clades. The branch leading to the outgroups *N. greeni* and *N. pretiosus* were removed to aid presentation, but joined the tree at the branch leading to the Northwest group. Bootstrap values are labelled at each node where they exceeded 70%. The topology presented is one of 5000 recovered (max trees = 5000) minimum length topologies.

**Figure S2.** Maximum clade credibility tree from Bayesian analysis based on 1420 bp of ND2 and ND4 DNA sequence from *Niveoscincus ocellatus* analysed using BEAST under a coalescence tree prior with a strict clock (normal distribution of rate variation with mean 1.52% sequence divergence per million years, and standard deviation 0.5% sequence divergence). Branch lengths are scaled proportional to time, as indicated by the scale bar. Where the sampling locality of an individual is not listed, it is the same as the first label that appears above.

**Figure S3.** TCS network for mitochondrial haplotypes (ND2 and ND4) from individuals of *Niveoscincus ocellatus*. Inferred haplotypes are indicated as bars on the links between observed haplotypes (circles). Circle fill patterns indicate localities of haplotypes with respect to the three mitochondrially-defined regions. Numbers correspond to explicit sampling localities of alleles as defined in Table S4. Broader shading defines geographically clustered haplotypes. Note that not all individuals depicted in the Bayesian Inference tree (Figure 2) are represented in this network owing to the missing data thresholds employed during network construction.
